# Comparison of keratoplasty outcomes at the scar versus edema stages of keratoconus

**DOI:** 10.5935/0004-2749.2023-0144

**Published:** 2024-03-27

**Authors:** Yingxin Chen, Zhida You, Cuiyu Wang, Ruiyao Gao, Kai Zhang

**Affiliations:** 1 Department of Ophthalmology, General Hospital of Northern Theater Command, Shenyang, China

**Keywords:** keratoconus, Edema, Cicatrix, keratoplasty, penetrating, Corneal transplantation, Astigmatism, Corneal endothelial cell loss, Endothelial cells

## Abstract

**Purpose:**

To assess the outcomes of deep anterior lamellar keratoplasty or penetrating keratoplasty at the scar and the edema stages.

**Methods:**

Forty-five patients (45 eyes) with keratoconus scar stage (scar group, n=26; penetrating keratoplasty a subgroup, n=7; deep anterior lamellar keratoplasty b subgroup, n=19) and keratoconus edema stage (edema group, n=19; penetrating keratoplasty c subgroup, n=12; deep anterior lamellar keratoplasty d group, n=7) who received penetrating keratoplasty or deep anterior lamellar keratoplasty from 2000 to 2022 were retrospectively studied. At 1, 6, and 12 months after surgery, the best-corrected visual acuity, astigmatism, spherical equivalent, corneal endothelial cell density, and complications were analyzed.

**Results:**

The best-corrected visual acuity and average corneal endothelial cell loss rate were not significantly different between the scar and edema groups (p>0.05). At 6 and 12 months after surgery, the astigmatism and spherical equivalent in the scar group were significantly lower than those in the edema group (p<0.05). The spherical equivalent of the deep anterior lamellar keratoplasty b subgroup was lower than that of the penetrating keratoplasty a subgroup in the scar group 6 months after surgery (p<0.05). In the edema group, there was no significant difference in spherical equivalent between subgroups (p>0.05). There were no significant differences in best-corrected visual acuity and astigmatism between subgroups within the two groups (p>0.05). In comparison to the scar group, the edema group experienced more complications. According to a survival analysis, there was no statistically significant difference between the scar group and the edema group regarding the progression of vision.

**Conclusions:**

In terms of the outcomes and prognosis for vision after keratoplasty with edema stage and scar stage, deep anterior lamellar keratoplasty may be as effective as penetrating keratoplasty.

## INTRODUCTION

Keratoconus is a dilated corneal disorder in which the local corneal stroma becomes thinner, the central part of the cornea bulges forward, becomes tapered, and produces extremely irregular astigmatism^(^1^,^2^)^. The disease can affect anyone between the ages of 15 and 20 but most typically strikes young people between the ages of 9 and 40. It is generally believed that the disease progresses more quickly the younger the onset. Acute corneal edema is a keratoconus condition that results from the Descemet membrane rupture that allows aqueous humor to enter the stroma and epithelial cells of the cornea^(^3^,^4^)^. After 3 months, the edema usually resolves, but scarring on the cornea frequently persists and impairs vision.

Keratoplasty can be effectively treated with keratoconus. It mainly consists of deep anterior lamellar keratoplasty (DALK) and penetrating keratoplasty (PKP)^(^2^)^. PKP is the most popular procedure for treating advanced keratoconus^(^5^)^, although there are several limitations, including postoperative endothelial rejection, corneal endothelial decompensation, and other complications^(^6^)^. In contrast to PKP, this surgical procedure offers the advantage of preventing corneal endothelial rejection in the future and preserving host endothelial cells during operation^(^7^)^. However, when DALK is used to treat advanced keratoconus, the severely dilated cornea increases the risk of intraoperative descemet membrane perforation and postoperative complications in patients with double anterior chambers^(^8^)^.

Edematous keratoconus is rarely treated with keratoplasty. However, Jacob S et al. showed that keratoconus can be treated in a modified DALK procedure to restore corneal structure and transparency^(^9^)^. The advantage of performing keratoplasty during the edema stage is that there is no need to wait 4-8 weeks for the corneal edema to subside^(^10^)^. This can reduce the risk of corneal rupture and infection during the edema stage^(^11^)^, reduce the formation of new blood vessels^(^12^)^, and reduce the risk of transplant rejection. However, further evidence is required to demonstrate its feasibility and safety. This study examined the outcomes of two keratoconus transplantation methods at the scar and edema stages.

## METHODS

### Patients

**Figure 1:**
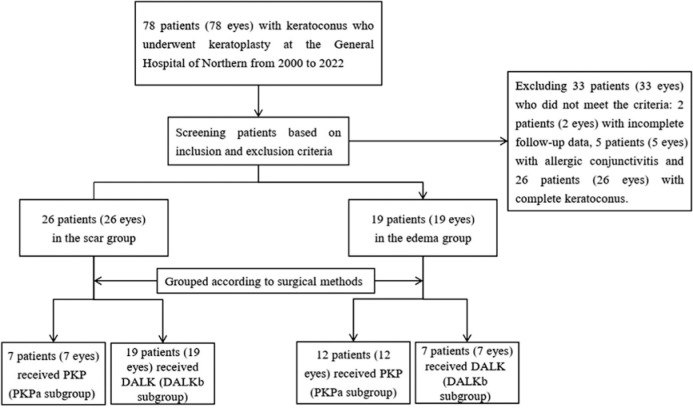
Organizational chart reflecting the grouping and exclusion criteria.

The data of patients with keratoconus at the scar or edema stage who underwent keratoplasty (PKP or DALK) at the General Hospital of Northern Theater Command during 2000-2022 were retrospectively collected. The patients were divided into the scar group (PKPa subgroup and DALKb subgroup) and edema group (PKPc subgroup and DALKd subgroup) (Figure 1). This study was approved by the Ethics Committee of the General Hospital of Northern Theater Command (Y(2022)134) and conducted in accordance with the Declaration of Helsinki. Informed consent of the patients was waived off owing to the retrospective nature of the study.

The study inclusion criteria were as follows: (1) the patient was diagnosed with keratoconus based on his medical history, slit-lamp microscopy (the central stroma was obviously thinner, with conical processes, Fleisher rings, Vogt lines, corneal stroma scars, etc) and corneal topographic map; (2) progressive vision loss in the affected eye; (3) corneal central curvature >55.0 D; (4) the postoperative follow-up time was >12 months. In the Edema Group, Descemet membrane rupture was observed under slit-lamp microscope. The exclusion criteria were as follows: (1) patients with secondary keratoconus; (2) patients with a history of internal eye surgery; (3) patients with ocular surface diseases such as dry eye or allergic conjunctivitis; (4) patients with other primary diseases that may affect their vision before surgery, such as congenital corneal leucoma, cataract, glaucoma, etc; (5) pregnant patients during the perioperative or follow-up period; (6) patients with a history of diabetes, hypertension, and hyperlipidemia; (7) patients on medications that affect their vision; (8) patients whose follow-up data were incomplete or lost follow-up.

### Surgical method

#### Preoperative routine treatment

The same experiences corneal transplant surgeons operate on all patients. Prior to the surgery, all patients received standard preoperative treatment for the inner eye. All procedures were carried out under local anesthesia. With the use of proparacaine hydrochloride eye drops, topical anesthesia was administered. Retrobulbar block anesthesia, peribulbar anesthesia, and orbicularis oculi block anesthesia were administered with 10 ml of an equal mixture of 2% lidocaine and 0.75% ropivacaine.

#### PKP

To obtain the pupil diameter down to <1 min, pilocarpine eye drops were administered 30 min prior to the PKP procedure. They were applied once every 5 min. The eyeball needs to be softened for 15 min prior to PKP. The eyelid was opened with an eye speculum, and the center of the cornea and 16-needle suture points were marked with methylene blue. A 7.5-8.5 mm trephine was used to create the implant holes. The holes should be made with the edges as neat as possible. A trephine that was 0.25 mm larger than the diameter of the implant hole was used to cut the graft from the inner skin of the donor. The grafts were sutured to the implant bed using 10-0 nylon sutures with 16 interrupted sutures.

#### DALK

The eyelid was opened with an eye speculum, and the center of the cornea and 16-needle suture points were marked with methylene blue. A sharp blade and an iris restorer cut through the anterior matrix after drilling the vacuum negative pressure trephine to a depth of roughly 1/2 of the implant bed. A few bubbles were injected into the anterior chamber using a 1 mL syringe to produce a “small bubble” after a 15-degree knife was used to puncture it. Using a 25G needle, immediately injected air into the stroma of the cornea to form a “big bubble” by pushing in the direction of the anterior chamber bubble. The center of the “big bubble” was cut, and a viscoelastic agent was injected into the bulla from this incision. The upper layer of the stromal bulla was divided into four quadrants by corneal scissors. The post-elastic membrane was revealed, the matrix tissue in four quadrants was cut off, and the implant bed was washed with a balanced salt solution. Select a trephine with an adequate diameter to drill the graft. Similar to PKP, the graft was sutured.

#### Postoperative management

To stabilize intraocular pressure, all patients received an intravenous infusion of 20% mannitol 250 ml twice daily and methylzolamide acetate 25 mg orally. The dosage was either reduced or discontinued depending on how the intraocular pressure changes. After the procedure, tobramycin and dexamethasone eye ointment were administered and bandaged. The dressing was changed every day until the eyes started to drop. After eye drops, they received tobramycin and dexamethasone eye ointment once at night. Levofloxacin eye drops four times a day. Deproteinized calf blood extract eye gel three times daily, and so forth. Prednisolone acetate ophthalmic suspension was administered three times daily for 2 weeks following surgery, along with levofloxacin eye drops four times daily, cyclosporine eye drops twice daily, and tobramycin and dexamethasone eye ointment once a night. The use and dosage of the drug were adjusted to fit the circumstances.

### Observation index

Each patient was followed up for a minimum of 1 year. The data collected included BCVA, astigmatism, spherical equivalent, corneal endothelial cell density, and complications at 1, 6, and 12 months following surgery. Visual acuity was assessed using the standard Snellen chart. For statistical analysis, the data were converted into logarithms of the minimum angle of resolution (logMAR) units. The spherical and cylindrical degrees were measured automatically with a refractor, and the spherical equivalent degree was calculated using the formula SEq = S + ½ Q^(^13^)^. The average rate of endothelial cell loss at 6 and 12 months following surgery was calculated based on 1 month following surgery.

### Statistical analysis

Data were analyzed using the IBM Statistical Package for Social Sciences Statistics 26.0. Measurement data were represented as mean ± standard deviation. Counting data were analyzed using the χ^^2^^ test. Paired sample t-tests were used to compare preoperative and postoperative data from the same operation method in each group for measurement data with normal distribution, and independent t-tests were used to compare data between the Edema Group and the Scar Group and between each subgroup. Nonnormally distributed data was analyzed using the non-parametric rank sum test. Patients in the scar and edema stages after corneal transplantation were compared for visual acuity recovery using the Kaplan-Meier survival curve and log-rank test. P-value <0.05 was considered statistically significant.

When α=0.05, and the non-inferiority margin value was 0.1, the non-inferiority test of two independent samples^(^14^)^ revealed that the Scar Group and the test efficiency of the Edema Group were 0.8536, β=0.1464. The non-inferiority efficacy test in the Scar Group and the Edema Group, α=0.05, the non-inferiority margin value was 0.15, the efficacy in the Scar Group was 0.8470, β=0.1531. The effectiveness in the Edema Group was 0.8074, β=0.1926. It can be concluded that this study has sufficient test efficiency^(^15^)^.

## RESULTS

### Baseline data of patients

The study involved 45 patients (45 eyes), of which 26 patients (26 eyes) were in the Scar Group and 19 patients (19 eyes) were in the Edema Group. Seven patients (7 eyes) received PKP (PKPa subgroup), while 19 patients (19 eyes) received DALK (DALKb subgroup) from the Scar Group. In the Edema Group, PKP was administrated to 12 patients (12 eyes) (PKPc subgroup), and DALK was administered to 7 patients (7 eyes) (DALKd subgroup). Age, sex, preoperative best-corrected visual acuity, graft diameter, and implant bed diameter did not significantly differ between the two groups (p>0.05) (Table 1).

**Table 1:** Comparison of the demographic and preoperative details between the Scar Group and the Edema Group

	Scar Group (n=26)	Edema Group (n=19)	p-value
Age (years)^*^	24.50 (10)	21.00 (10)	0.170
**Sex (male/female)**	17/9	10/9	0.388
**Laterality (right/left)**	15/11	8/11	0.302
BCVA (LogMAR)^*^	1.85 (1.70)	2.70 (1.40)	0.177
Graft diameter (mm)^*^	7.75 (0.25)	7.75 (0.25)	0.787
Implant bed diameter (mm)^*^	7.50 (0.31)	7.50 (0.50)	0.428

* The data does not conform to normal distribution and is expressed by median. Independent sample Mann-Whitney U-test was applied for the test.

### Visual outcomes

The mean BCVA of patients in the Scar and the Edema Group was significantly higher than before surgery (p<0.05). At each time point, the two groups had no significant difference in mean BCVA. In the scar group, the mean BCVA was marginally higher than in the Edema Group (Table 2). At each time following surgery, there was no significant difference in the mean BCVA between the PKPa and DALKb subgroups in the Scar Group (p>0.05). At 1 to 6 months following surgery, the mean BCVA of the PKPa subgroup was marginally worse than that of the DALKb subgroup. At 12 months following surgery, the mean BCVA of the PKPa subgroup was marginally higher than that of the DALKb subgroup (Table 3). At each time point, there was no significant difference in mean BCVA between PKPc and DALKd subgroups (p>0.05). At 1 to 6 months following surgery, the mean BCVA of the PKPc subgroup was marginally higher than that of the DALKd subgroup. At 12 months, the mean BCVA of the PKPc subgroup was somewhat worse than that of the DALKd subgroup (Table 3).

**Table 2:** Comparison of the outcomes between the Scar Group and the Edema Group

	Time (month)	Scar Group (mean ± SD)	Edema Group (mean ± SD)	t	p-value
**BCVA**	1	0.43 ± 0.20	0.37 ± 0.20	0.942	0.351
	6	0.40 ± 0.16	0.37 ± 0.19	0.448	0.656
	12	0.24 ± 0.12	0.23 ± 0.12	0.187	0.853
**Astigmatism**	1	3.56 ± 0.68	4.28 ± 0.57	-3.756	0.001
	6	3.36 ± 0.66	4.02 ± 0.60	-3.419	0.001
	12	3.05 ± 0.70	3.61 ± 0.55	-2.901	0.006
**Spherical equivalent**	1	2.63 ± 0.74	3.03 ± 0.60	-1.896	0.065
	6	2.63 ± 0.68	3.41 ± 0.64	-3.935	<0.001
	12	2.44 ± 0.73	3.17 ± 0.59	-3.581	0.001

**Table 3:** Comparison of the outcomes in different operation groups

	Time (month)	Scar Group				Edema Group			
		PKPa (mean ± SD)	DALKb (mean ± SD)	t	p-value	PKPc (mean ± SD)	DALKd (mean ± SD)	t	p-value
BCVA	1	0.37 ± 0.28	0.45 ± 0.16	-0.918	0.368	0.38 ± 0.23	0.36 ± 0.17	0.265	0.794
	6	0.36 ± 0.22	0.41 ± 0.14	-0.663	0.514	0.38 ± 0.21	0.36 ± 0.17	0.188	0.853
	12	0.26 ± 0.15	0.23 ± 0.12	0.461	0.649	0.22 ± 0.13	0.26 ± 0.98	-0.697	0.496
Astigmatism	1	3.75 ± 0.54	3.49 ± 0.72	0.877	0.389	4.38 ± 0.52	4.11 ± 0.66	0.986	0.338
	6	3.54 ± 0.51	3.29 ± 0.71	0.834	0.413	4.10 ± 0.57	3.86 ± 0.66	0.863	0.400
	12	3.04 ± 0.47	3.05 ± 0.77	-0.054	0.957	3.60 ± 0.58	3.61 ± 0.54	-0.011	0.991
Spherical equivalent	1	2.97 ± 0.73	2.51 ± 0.72	1.407	0.172	3.04 ± 0.57	3.00 ± 0.69	0.142	0.889
	6	3.04 ± 0.42	2.45 ± 0.66	2.173	0.040	3.60 ± 0.58	3.07 ± 0.62	1.882	0.077
	12	2.79 ± 0.40	2.32 ± 0.79	1.493	0.148	3.35 ± 0.56	2.86 ± 0.54	1.896	0.075

### Postoperative astigmatism

When the astigmatism values of the scar group and Edema Group were compared at 1 month, 6 months, and 12 months after surgery (p<0.05), the astigmatism value of the Scar Group was significantly lower than that of the Edema Group (Table 2). At each time point in the Scar Group, there was no significant difference in astigmatism between the PKPa and the DALKb subgroups (p>0.05). At 1 and 6 months following surgery, postoperative astigmatism in the Scar Group was marginally higher in the PKPa Group compared to the DALKb Group. Postoperative astigmatism in the PKPa Group in the Scar Group was marginally reduced at 12 months postoperatively than in the DALKb Group (Table 3). At each time point in the Edema Group, there was no significant difference in astigmatism between the PKPc and DALKd subgroups (p>0.05). Six months following surgery, the astigmatism in the PKPc Group was somewhat higher than that in the DALKd Group. Astigmatism in the PKPc Group was slightly lower than in the DALKd Group at 12 months following surgery (Table 3).

### Spherical equivalent

A month after surgery, there was no significant difference between the Scar and Edema Groups (p>0.05). The spherical equivalent of the Scar Group was significantly lower than that of the Edema Group 6 months and 12 months following surgery (p<0.05) (Table 2). The spherical equivalent of the PKPa Group in the Scar Group was significantly higher than that in the DALKb Group (p<0.05) 6 months after surgery. No statistically significant difference occurred in the 1 and 12 months following surgery. In the Edema Group, there was no statistically significant difference between the PKPc and the DALKd subgroups at any given time (Table 3).

### Postoperative corneal endothelial cell density

There was no significant difference in the mean endothelial cell loss rate between the Scar Group and the Edema Group 6 and 12 months following surgery (p>0.05) (Table 4). At 6 and 12 months following surgery, the mean endothelial cell loss rate in the PKPa subgroup was significantly higher than in the DALKb subgroup (p<0.001). At 6 and 12 months following surgery, the mean endothelial cell loss rate in the PKPc subgroup was significantly higher than that in the DALKd subgroup at 6 and 12 months after operation (p<0.001) (Table 5).

**Table 4:** Comparison of cells/mm^2^ (%) of endothelial cell loss between the Scar Group and Edema Group

Time (month)	Scar Group (mean ± SD)	Edema Group (mean ± SD)	t	p-value
**1**	2693.42 ± 279.83 (100)	2583.47 ± 346.09 (100)	1.178	0.245
**6**	2545.50 ± 295.51 (4.56 ± 3.81)^*^	2403.74 ± 385.23 (7.47 ± 5.21)^*^		0.168
**12**	2405.35 ± 327.99 (8.92 ± 9.56)^*^	2224.95 ± 413.12 (15.16 ± 11.14)^*^		0.093

* The data does not conform to normal distribution and is expressed by median. Independent sample Mann-Whitney U-test was used for the test.

**Table 5:** Comparison of cells/mm^2^ (%) of endothelial cell loss in different operation groups

Time (month)	Scar Group				Edema Group			
	PKPa (mean ± SD)	DALKb (mean ± SD)	t	p-value	PKPc (mean ± SD)	DALKd (mean ± SD)	t	p-value
1	2572.71 ± 336.92 (100)	2737.89 ± 251.33 (100)	-1.357	0.187	2441.08 ± 303.78 (100)	2827.57 ± 282.99 (100)	-2.740	0.014
6	2325.86 ± 298.29 (9.56 ± 1.42)	2626.42 ± 256.82 (4.12 ± 1.02)	10.826	<0.001	2215.67 ± 305.99 (9.37 ± 2.31)	2726.14 ± 285.57 (3.63 ± 1.06)	6.156	<0.001
12	2098.43 ± 304.80 (18.55 ± 1.48)	2518.42 ± 261.37 (8.11 ± 1.50)	16.112	<0.001	1998.42 ± 298.37 (18.34 ± 2.90)	2613.29 ± 265.00 (7.59 ± 0.33)	9.645	<0.001

### Complications

In the Scar Group, complications affected 15 eyes (57.69%), while in the Edema Group, complications affected 17 eyes (89.47%) (Table 6). There were only intraoperative complications in the DALKb and the DALKd subgroups.

**Table 6:** Complications of the Scar Group and Edema Group

Complication	Scar Group (n=26)			Edema Group (n=19)		
	PKPa (n=7)	DALKb (n=19)	Total	PKPc (n=12)	DALKd (n=7)	Total
**Intraoperation** **Descemet membrane microperforation**		1 (5.26%)	1 (3.85%)		1 (14.30%)	1 (5.26%)
**Postoperation** **Shallow anterior chamber**	2 (28.60%)		2 (7.69%)	2 (16.7%)		2 (10.52%)
**Double anterior chamber**		3 (15.80%)	3 (11.54%)		2 (28.60%)	2 (10.52%)
**High intraocular pressure**	3 (42.90%)	1 (5.26%)	4 (15.38%)	4 (33.30%)	1 (14.30%)	5 (26.32%)
**Secondary glaucoma**				1 (8.33%)	1 (14.30%)	2 (10.52%)
**Graft rejection**	2 (28.60%)	2 (10.53%)	4 (15.38%)	3 (25.00%)	1 (14.30%)	4 (21.05%)
**Secondary cataract**	1 (14.30%)		1 (3.85%)	1 (8.33%)		1 (5.26%)
**Total**	8	7	15 (57.69%)	11	6	17 (89.47%)

### Survival analysis

**Figure 2:**
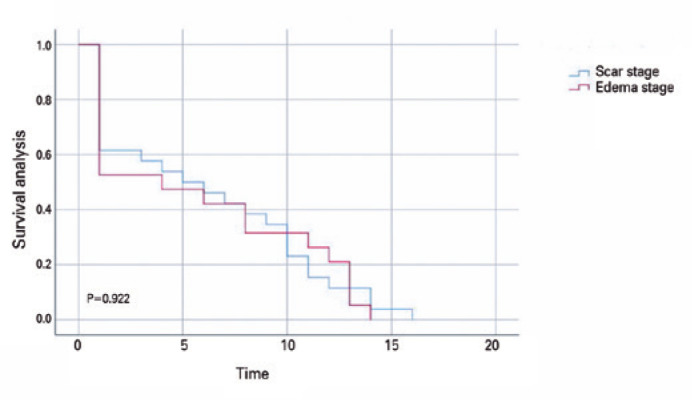
Kaplan-Meier survival curves of keratoplasty in keratoconus patients at different stages.

Excluding loss of follow-up and changes in visual acuity due to reasons unrelated to disease and surgery, the time from postoperative to LogMAR visual acuity ≤0.3 was measured. There were 45 patients, with 26 in the Scar Group and 19 in the Edema Group. According to the log-rank test, there was no statistically significant difference between the prognosis of patients at different stages (p=0.922). The Kaplan-Meier curve demonstrates that patients in the Scar and Edema Groups have significantly improved vision following corneal transplantation. LogMAR of all patients reached 0.3 within 20 months, and 50% of patients in the Scar and the Edema Groups reached this visual acuity at 5 and 4 months, respectively. This indicates that the visual acuity of the Edema Group improved faster than that of the Scar Group (Figure 2).

## DISCUSSION

Keratoconus^(^16^)^ can be effectively treated with keratoplasty. Keratoconus at the edema stage is not a contraindication for keratoplasty, given the development and maturation of the process. Several inferences can be reached from our analysis. A prognosis identical to that in the scar stage is obtained after keratoplasty in the keratoconus edema stage.

According to Jacob et al., postoperative eyesight following DALK treatment in the edema stage was much better than before surgery^(^9^)^. This study discovered that postoperative visual acuity was significantly improved when PKP or DALK was used to treat keratoconus in the scar stage and edema stage. After surgery, long--term visual acuity recovered to more than 0.3; in the Edema Group, it did so much faster than in the Scar Group. Furthermore, there was no significant difference between the Scar and the Edema Groups in the long-term endothelial cell loss rate. Edematous and cicatricial keratoplasty have no impact on endothelial cell loss. After PKP, there was a significantly higher rate of long-term endothelium loss than after DALK. This was consistent with the study of Cheng et al.^(^17^)^, which showed that endothelial cell loss persisted following PKP surgery and was significantly higher than that of DALK surgery. The operation of DALK is challenging and fraught with hazards like endothelial perforation since the rupture of the descemet membrane of the keratoconus in the acute stage is accompanied by corneal edema.

Due to the higher corneal curvature in the edema stage, patients in the Edema Group in this study had significantly higher postoperative astigmatism than those in the Scar Group. Drilling and cutting caused the wound edge to be more prone to tilting, and when the incision healed, ring scars formed, increasing the astigmatism. Additionally, the high corneal curvature after corneal transplantation may result in postoperative graft folds^(^18^,^19^)^, increasing astigmatism. The research findings of Yu et al.^(^20^)^ indicated that the choice of two surgical methods does not alter the astigmatism results and that there was no significant difference in postoperative astigmatism between PKP and DALK.

The incidence of intraoperative complications was slightly higher in the Edema Group than in the Scar Group. Both groups experienced intraoperative problems while the deep matrix was being separated and the descemet membrane was being stripped by DALK. According to other research, microperforation of the descemet membrane^(^21^,^22^)^ is the primary intraoperative complication that occurs more frequently in DALK than in PKP.

Compared to the Scar Group, the incidence of postoperative transplant rejection was slightly higher in the Edema Group. Localized vascular endothelial growth factor releases occur in the eye when keratoconus reaches the edema stage. The risk of corneal neovascularization increases^(^12^)^ with edema time, increasing the chances of postoperative rejection. Janiszewska-Bil et al.^(^23^)^, Funnell et al.^(^24^)^ and Watson et al.^(^25^)^ revealed no significant diffe-rence in long-term vision recovery between the two surgical procedures. However, because PKP has endothelial rejection, it may result in further complications. The rejection reaction in the Edema Group was higher than that in the Scar Group in this study because there were more patients receiving PKP in the Edema Group than in the Scar Group, and because the risk of rejection following PKP was higher^(^26^,^27^)^. Overall, we can say that keratoplasty is generally safe during the scar stage and edema stages.

This study has several limitations. This study has a limited sample size and is a non-random retrospective study. Due to corneal scarring or edema in patients, preoperative baseline optometry data were unavailable, making it difficult to discuss whether and how baseline data affected prognosis. The impact of different intraoperative and postoperative complications on the long--term recovery of patients was not further investigated.

Regarding the results and prognosis for vision following keratoplasty with edema and scar stage, DALK might be just as effective as PKP. Although the visual acuity improved faster during the edema stage, there were more complications than during the scar stage.

## References

[1] Wajnsztajn D, Solomon A. (2021). Vernal keratoconjunctivitis and keratoconus. Curr Opin Allergy Clin Immunol.

[2] Santodomingo-Rubido J, Carracedo G, Suzaki A, Villa-Collar C, Vincent SJ, Wolffsohn JS. (2022). Keratoconus: an updated review. Cont Lens Anterior Eye.

[3] Maharana PK, Sharma N, Vajpayee RB. (2013). Acute corneal hydrops in keratoconus. Indian J Ophthalmol.

[4] García de Oteyza G, Bregliano G, Sassot I, Quintana L, Rius C, García-Albisua AM. (2021). Primary surgical options for acute corneal hydrops: A
review. Eur J Ophthalmol.

[5] Seitz B, Daas L, Hamon L, Xanthopoulou K, Goebels S, Spira-Eppig C (2021). Stage-appropriate treatment of keratoconus. Ophthalmologe.

[6] Kim KH, Choi SH, Ahn K, Chung ES, Chung TY. (2011). Comparison of refractive changes after deep anterior lamellar
keratoplasty and penetrating keratoplasty for keratoconus. Jpn J Ophthalmol.

[7] Reinhart WJ, Musch DC, Jacobs DS, Lee WB, Kaufman SC, Shtein RM. (2011). Deep anterior lamellar keratoplasty as an alternative to
penetrating keratoplasty a report by the American Academy of
Ophthalmology. Ophthalmology.

[8] Feizi S, Javadi MA, Khajuee-Kermani P, Jafari R. (2017). Repeat Keratoplasty for Failed Deep Anterior Lamellar
Keratoplasty in Keratoconus: Incidence, Indications, and
Outcomes. Cornea.

[9] Jacob S, Narasimhan S, Agarwal A, Sambath J, Umamaheshwari G, Saijimol AI. (2018). Primary Modified Predescemetic Deep Anterior Lamellar
Keratoplasty in Acute Corneal Hydrops. Cornea.

[10] Fuentes E, Sandali O, El Sanharawi M, Basli E, Hamiche T, Goemaere I (2015). Anatomic predictive factors of acute corneal hydrops in
keratoconus: an optical coherence tomography study. Ophthalmology.

[11] Rubsamen PE, McLeish WM. (1991). Keratoconus with acute hydrops and perforation. Brief case
report. Cornea.

[12] Sharif Z, Sharif W. (2019). Corneal neovascularization: updates on pathophysiology,
investigations & management. Rom J Ophthalmol.

[13] Mohan K, Sharma SK. (2022). Clinically significant changes in the spherical equivalent
hyperopia in patients with refractive accommodative
esotropia. J Clin Ophthalmol Res.

[14] Julious SA. (2004). Sample sizes for clinical trials with normal data. Stat Med.

[15] Khan Z, Milko J, Iqbal M, Masri M, Almeida DRP. (2016). Low power and type II errors in recent ophthalmology
research. Can J Ophthalmol J Can Ophtalmol.

[16] Choi JH, Jeng BH. (2022). Indications for keratoplasty in management of corneal
ectasia. Curr Opin Ophthalmol.

[17] Cheng YY, Visser N, Schouten JS, Wijdh RJ, Pels E, van Cleynenbreugel H (2011). Endothelial cell loss and visual outcome of deep anterior
lamellar keratoplasty versus penetrating keratoplasty: a randomized
multicenter clinical trial. Ophthalmology.

[18] Shi W, Li S, Gao H, Wang T, Xie L. (2010). Modified deep lamellar keratoplasty for the treatment of
advanced-stage keratoconus with steep curvature. Ophthalmology.

[19] Kumar M, Shetty R, Khamar P, Vincent SJ. (2020). Scleral lens-induced corneal edema after penetrating
keratoplasty. Optom Vis Sci.

[20] Yu AC, Mattioli L, Busin M. (2020). Optimizing outcomes for keratoplasty in ectatic corneal
disease. Curr Opin Ophthalmol.

[21] Keane M, Coster D, Ziaei M, Williams K. (2014). Deep anterior lamellar keratoplasty versus penetrating
keratoplasty for treating keratoconus. Cochrane Database Syst Rev.

[22] Händel A, Lüke JN, Siebelmann S, Franklin J, Roters S, Matthaei M (2022). Outcomes of deep anterior lamellar keratoplasty and penetrating
keratoplasty in keratoconic eyes with and without previous
hydrops. Graefes Arch Clin Exp Ophthalmol.

[23] Janiszewska-Bil D, Czarnota-Nowakowska B, Krysik K, Lyssek-Boroń A, Dobrowolski D, Grabarek BO (2021). Comparison of long-term outcomes of the lamellar and penetrating
keratoplasty approaches in patients with keratoconus. J Clin Med.

[24] Funnell CL, Ball J, Noble BA. (2006). Comparative cohort study of the outcomes of deep lamellar
keratoplasty and penetrating keratoplasty for keratoconus. Eye (Lond).

[25] Watson SL, Ramsay A, Dart JK, Bunce C, Craig E. (2004). Comparison of deep lamellar keratoplasty and penetrating
keratoplasty in patients with keratoconus. Ophthalmology.

[26] Ziaei M, Vellara HR, Gokul A, Ali NQ, McGhee CN, Patel DV. (2020). Comparison of corneal biomechanical properties following
penetrating keratoplasty and deep anterior lamellar keratoplasty for
keratoconus. Clin Exp Ophthalmol.

[27] Aytekin E, Pehlivan SB. (2021). Corneal cross-linking approaches on keratoconus
treatment. J Drug Deliv Sci Technol.

